# Phenotyping Cardiopulmonary Exercise Limitations in Chronic Obstructive Pulmonary Disease

**DOI:** 10.3389/fphys.2022.816586

**Published:** 2022-02-15

**Authors:** Jinelle Gelinas, Megan Harper, John Sasso, Stephen Wright, Bernie Melzer, Gloria Agar, Jordan Guenette, Gregory duManoir, Michael Roman, J. Douglass Rolf, Neil Eves

**Affiliations:** ^1^Centre for Heart, Lung and Vascular Health, University of British Columbia, Kelowna, BC, Canada; ^2^Interior Health Authority, Kelowna General Hospital, Kelowna, BC, Canada; ^3^Department of Physical Therapy and Centre for Heart Lung Innovation, University of British Columbia and St. Paul’s Hospital, Vancouver, BC, Canada; ^4^Faculty of Medicine, University of Calgary, Calgary, AB, Canada; ^5^Faculty of Medicine, University of British Columbia, Vancouver, BC, Canada

**Keywords:** COPD, cardiopulmonary exercise testing, clinical exercise physiology, exercise limitations, exercise prescription

## Abstract

**Background:**

Exercise limitation in chronic obstructive pulmonary disease (COPD) is commonly attributed to abnormal ventilatory mechanics and/or skeletal muscle function, while cardiovascular contributions remain relatively understudied. To date, the integrative exercise responses associated with different cardiopulmonary exercise limitation phenotypes in COPD have not been explored but may provide novel therapeutic utility. This study determined the ventilatory, cardiovascular, and metabolic responses to incremental exercise in patients with COPD with different exercise limitation phenotypes.

**Methods:**

Patients with COPD (*n* = 95, FEV_1_:23–113%pred) performed a pulmonary function test and incremental cardiopulmonary exercise test. Exercise limitation phenotypes were classified as: ventilatory [peak ventilation (V_Epeak_)/maximal ventilatory capacity (MVC) ≥ 85% or MVC-V_Epeak_ ≤ 11 L/min, and peak heart rate (HR_peak_) < 90%pred], cardiovascular (V_Epeak_/MVC < 85% or MVC-V_Epeak_ > 11 L/min, and HR_peak_ ≥ 90%pred), or combined (V_Epeak_/MVC ≥ 85% or MVC-V_Epeak_ ≤ 11 L/min, and HR_peak_ ≥ 90%pred).

**Results:**

FEV_1_ varied within phenotype: ventilatory (23–75%pred), combined (28–90%pred), and cardiovascular (68–113%pred). The cardiovascular phenotype had less static hyperinflation, a lower end-expiratory lung volume and larger tidal volume at peak exercise compared to both other phenotypes (*p* < 0.01 for all). The cardiovascular phenotype reached a higher V_Epeak_ (60.8 ± 11.5 L/min vs. 45.3 ± 15.5 L/min, *p* = 0.002), cardiopulmonary fitness (VO_2peak_: 20.6 ± 4.0 ml/kg/min vs. 15.2 ± 3.3 ml/kg/min, *p* < 0.001), and maximum workload (103 ± 34 W vs. 72 ± 27 W, *p* < 0.01) vs. the ventilatory phenotype, but was similar to the combined phenotype.

**Conclusion:**

Distinct exercise limitation phenotypes were identified in COPD that were not solely dependent upon airflow limitation severity. Approximately 50% of patients reached maximal heart rate, indicating that peak cardiac output and convective O_2_ delivery contributed to exercise limitation. Categorizing patients with COPD phenotypically may aid in optimizing exercise prescription for rehabilitative purposes.

## Introduction

Chronic obstructive pulmonary disease (COPD) is a complex heterogenous condition with diverse clinical presentations and prognoses that cannot be entirely explained by differences in airflow limitation and dyspnea (Agusti et al., 2010; Casanova et al., 2011). As such, delineating clinical phenotypes in COPD is important to facilitate the prescription of targeted therapies to optimize clinical outcomes. Incremental cardiopulmonary exercise testing (CPET) is an important tool in the risk stratification of patients due to the integrative assessment of physiological responses that can help distinguish subgroups of patients with unique disease characteristics (Oga et al., 2003; Yoshimura et al., 2014; Neder et al., 2019), and may provide therapeutic utility beyond the severity of airflow obstruction.

In COPD, exercise limitation has classically been attributed to expiratory flow limitation causing an abnormal rise in lung volumes. In patients with greater static and/or dynamic hyperinflation, end-inspiratory lung volume (EILV) rises close to total lung capacity (TLC) during exercise and normal tidal volume (V_T_) expansion becomes mechanically constrained (Laveneziana et al., 2011; O’Donnell et al., 2012). The greater mechanical work associated with breathing at higher lung volumes and at a greater frequency increases inspiratory neural drive, while the ability to efficiently increase minute ventilation (V_E_) is reduced (O’Donnell et al., 2006, 2012; Ofir et al., 2008; Laveneziana et al., 2011; Guenette et al., 2014). The resulting imbalance ultimately leads to the sensation of dyspnea, early exercise cessation and an attenuated peak O_2_ consumption (VO_2peak_) (O’Donnell et al., 2006, 2012; Ofir et al., 2008; Laveneziana et al., 2011; Guenette et al., 2014).

It is intuitive that patients with COPD would be primarily limited by the pulmonary system; however, considerable evidence supports that all systems in the O_2_ cascade integratively contribute to the body’s inability to meet metabolic demand (Maltais et al., 2000; Puente-Maestu et al., 2009; Broxterman et al., 2020). Although rarely acknowledged, a number of patients with COPD reach age-predicted maximal heart rate (HR_max_) with or without a ventilatory reserve during incremental CPET (Babb et al., 1991; Plankeel et al., 2005). In health, VO_2peak_ is predominantly limited by the cardiovascular system; stroke volume plateaus at ~50%VO_2peak_ and cardiac output cannot increase further once HR_max_ is reached (Astrand et al., 1964; Higginbotham et al., 1986; Plotnick et al., 1986). As such, the observation that certain patients with COPD reach HR_max_ suggests that cardiac output and convective O_2_ delivery are maximized, indicating a significant cardiovascular contribution to exercise limitation. However, whether the integrative physiological exercise responses [e.g., lung volumes, exertional symptoms, VO_2peak_, and maximum workload (W_max_)] differ in patients who have different cardiopulmonary exercise limitations has not been studied. Thus, this study aimed to determine the distinct ventilatory, cardiovascular, and metabolic responses to incremental CPET in patients with COPD who presented with either a ventilatory, cardiovascular, or combined (reach both ventilatory and cardiovascular criteria) exercise limitation. We hypothesized that the cardiovascular limited phenotype would have the least amount of static and dynamic hyperinflation, and thus the greatest V_T_ expansion during exercise. Consequently, VO_2peak_ and W_max_ would be higher in the cardiovascular phenotype compared to the ventilatory and combined phenotypes.

## Materials and Methods

**Figure 1:**
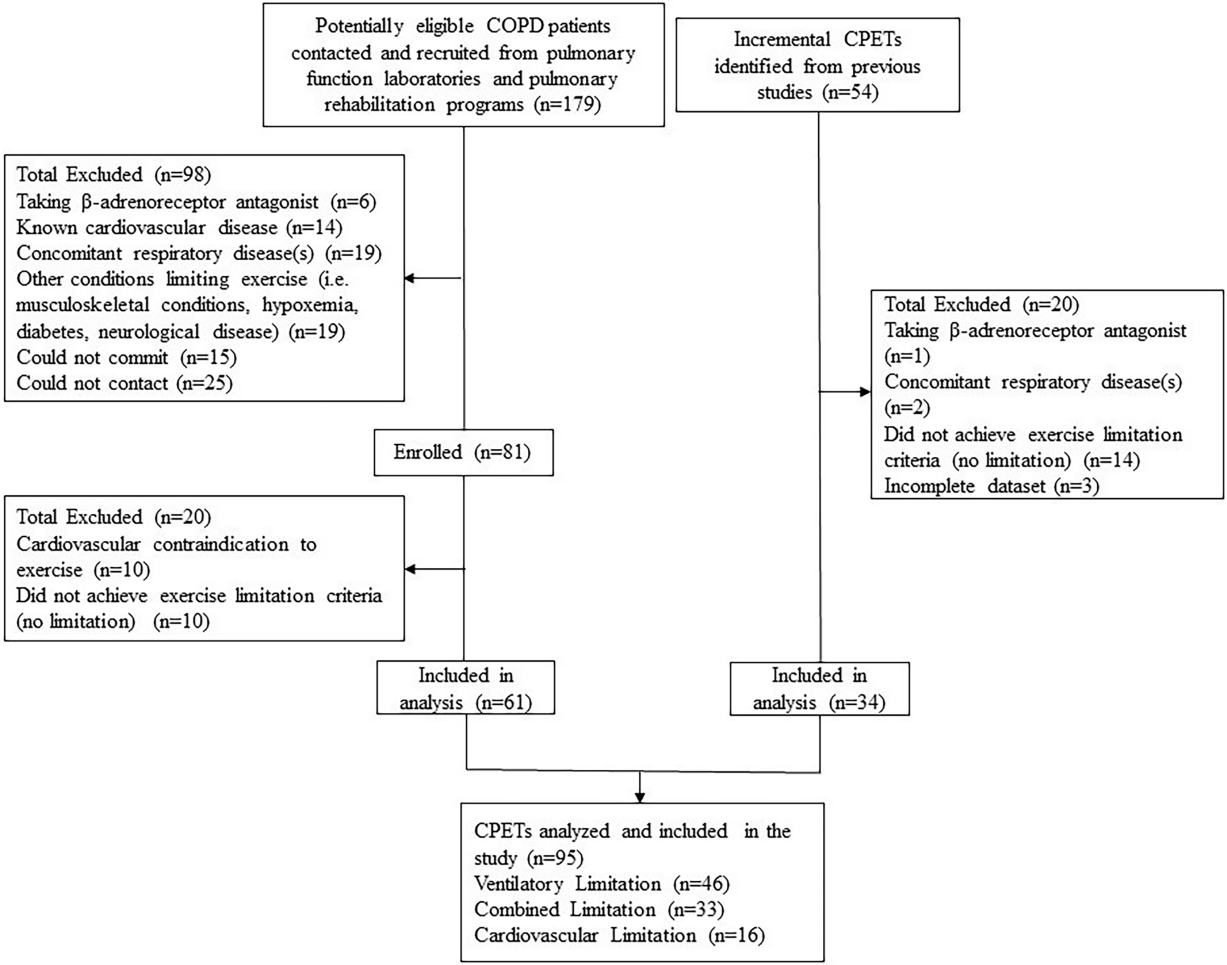
Study participant flow.

Stable individuals with physician confirmed COPD [post bronchodilator forced expiratory volume in 1 s (FEV_1_)/forced vital capacity (FVC) < 0.7 and below the lower limit of normal (LLN); Culver et al., 2017] were included. Patients were excluded if they had recently experienced an exacerbation (<3 months), were taking a β-adrenoreceptor antagonist, had a concomitant condition that could influence exercise limitation (i.e., other respiratory condition, neuromuscular disease, diabetes, or hypoxemia), presented with a cardiovascular contraindication to exercise or did not achieve the predetermined exercise limitation criteria. Study participant flow is depicted in Figure 1. Testing was performed at the Universities of British Columbia (*n* = 55) and Calgary (*n* = 6), and identical protocols were used at both sites. Participants signed an informed consent form that had received approval from the University of British Columbia Clinical Research Ethics Board and the University of Calgary Conjoint Health Research Ethics Board. Additionally, 34 incremental CPETs previously conducted to screen for exercise contraindications in prior studies were retrospectively analyzed and included. While the submaximal exercise responses have never been published, some of the peak exercise responses (*n* = 22/34) have been published elsewhere (Davidson et al., 2012; Gelinas et al., 2017).

### Pulmonary Function and Exercise Testing

Pulmonary function (6200-Autobox; SensorMedics, CA, United States) was assessed according to the American Thoracic Society (ATS)/European Respiratory Society guidelines (Wanger et al., 2005; Graham et al., 2017, 2019). An incremental CPET was performed to symptom limitation on an electrically braked cycle ergometer (Ergoselect 200, SensorMedics GmbH, Bitz, Germany) with expired breath-by-breath gas analysis [V_max_-29C, SensorMedics, CA, United States (*n* = 89) or QuarkCPET, COSMED, Italy (*n* = 6)] according to ATS/American College of Chest Physicians (ACCP) guidelines (American Thoracic Society and American College of Chest Physicians, 2003). Following 5-min of stable resting ventilatory values, participants cycled unloaded for 1-min followed by an increase in 5–10 watts/min until symptom limitation. Oxyhemoglobin saturation (SpO_2_; Radical 7, Maximo, CA, United States) and heart rate (12-lead ECG; CardioSoftTM, GE-Healthcare, WI, United States) were monitored continuously. Exertional symptoms (modified 0–10 Borg Scale; Borg, 1982) and inspiratory capacity (IC; Yan et al., 1997) were measured every 2-min. VO_2peak_ and V_Epeak_ were selected as the highest 30-s average, while peak heart rate (HR_peak_) was the highest recorded. The V_E_-VCO_2_ slope and intercept were determined by plotting 30-s averages of V_E_ vs. VCO_2_ following the first minute of exercise until the respiratory compensation point, which was considered the lowest V_E_/VCO_2_ (nadir) before a consistent rise and confirmed by the modified Beaver plot (Wasserman et al., 1973; Beaver et al., 1986). If the respiratory compensation point could not be identified, all data were included and the lowest V_E_/VCO_2_ was considered the nadir. Exercise limitation was determined according to ATS/ACCP recommendations whereby maximal ventilatory capacity (MVC) was estimated as 35*FEV_1_ and age-predicted HR_max_ was calculated as 220-age (American Thoracic Society and American College of Chest Physicians, 2003). Phenotypes were classified as ventilatory (V_Epeak_/MVC ≥ 85% or MVC-V_Epeak_ ≤ 11 L/min, and HR_peak_ < 90%pred), cardiovascular (V_Epeak_/MVC < 85% or MVC-V_Epeak_ > 11 L/min, and HR_peak_ ≥ 90%pred), or combined (V_Epeak_/MVC ≥ 85% or MVC-V_Epeak_ ≤ 11 L/min, and HR_peak_ ≥ 90%pred).

### Statistical Analysis

Normality was assessed with the Shapiro–Wilk test. Parametric data were analyzed with a one-way ANOVA and Tukey HSD *post hoc* at rest, 40 W (isoload-1) and peak exercise. Differences between the cardiovascular and combined phenotypes at 60 W (isoload-2) were assessed with an independent *t*-test. Isoloads represented the highest workload achieved by ≥90% of patients in each phenotype. Appropriate non-parametric tests were performed as needed. Data are presented as mean ± SD. Utilizing data from our laboratory, it was anticipated that 55, 35, and 10% of COPD patients would be ventilatory, combined, or cardiovascular limited, respectively. Assuming similar proportions, a minimum difference of the change in IC (ΔIC) between groups of 200 ml, a SD of 300 ml, a *β* = 0.8, and a two-tailed *α* = 0.017 (to correct for multiple comparisons), 69 participants was the minimum required.

## Results

**Figure 2:**
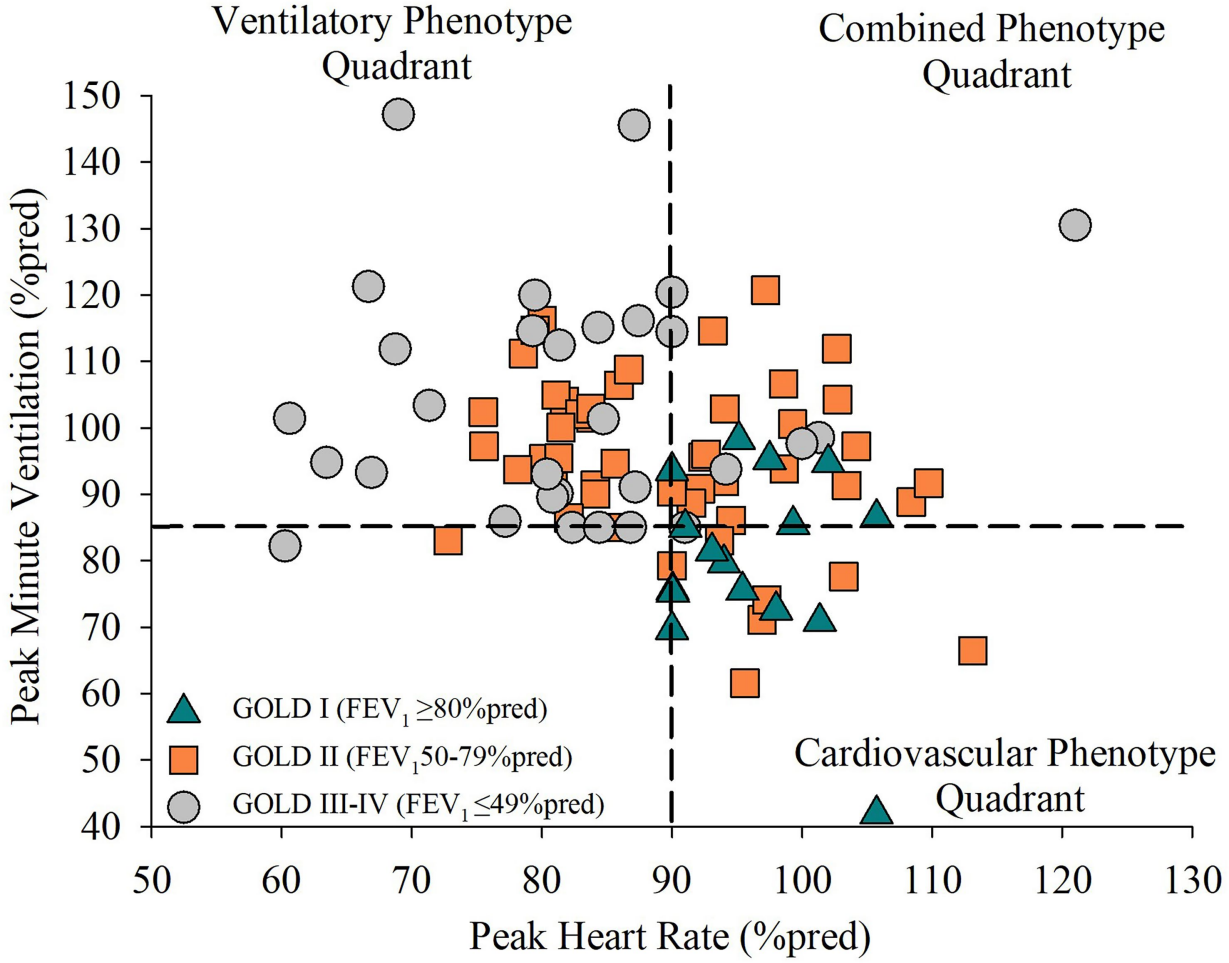
The distribution of airflow limitation severity in patients with chronic obstructive pulmonary disease (COPD) who have a ventilatory, combined, or cardiovascular exercise limitation phenotype. Phenotype quadrants are differentiated by a dash horizontal and vertical line representing the classification criteria for a ventilatory limitation (V_Epeak_/MVC ≥ 85%) and cardiovascular limitation (HR_peak_ ≥ 90%pred), respectively. GOLD severity is represented by the symbols to show the range of airflow limitation severity within each phenotype. GOLD I (mild airflow limitation) is represented by triangles. GOLD II (moderate airflow limitation) is represented by squares. GOLD III–IV (severe to very severe airflow limitation) is represented by circles.

Ninety-five patients were included (Figure 1). Phenotype characteristics are presented in Table 1. Forty-eight, 35, and 17% of patients were classified with a ventilatory, combined, or cardiovascular phenotype, respectively. Age, body mass index, and smoking history were not different between phenotypes. The ventilatory phenotype included more males and reported a higher MRC dyspnea compared to both other phenotypes. FEV_1_/FVC and FEV_1_ were significantly different between phenotypes with a wide range within each: ventilatory (FEV_1_:23–75%pred), combined (28–90%pred), and cardiovascular (68–113%pred; Figure 2). The cardiovascular phenotype had a lower residual volume (RV)/TLC ratio compared to both other phenotypes, while RV and functional residual capacity (FRC) were lower and IC/TLC was greater compared to the ventilatory phenotype.

**Table 1:** Phenotype characteristics and pulmonary function.

Variable	Ventilatory (*n* = 46)	Combined (*n* = 33)	Cardiovascular (*n* = 16)	ANOVA *p*-value
Males:females	27:19	17:16	8:8	0.75^a^
Age (years)	68 ± 7	71 ± 7	68 ± 8	0.24
Height (m)	1.69 ± 0.09	1.68 ± 0.11	1.70 ± 0.09	0.77
Body mass index (kg/m^2^)	27.1 ± 5.9	27.5 ± 3.6	26.3 ± 3.4	0.61
Smoking history (pk yr)	38 ± 23	33 ± 19	26 ± 18	0.11
MRC dyspnea score	3 ± 1^*^^†^	2 ± 1	2 ± 1	<0.01
FEV_1_ (L)	1.34 ± 0.45^*^^†^	1.66 ± 0.45^‡^	2.43 ± 0.59	<0.01
FEV_1_ (% pred)	49 ± 13^*^^†^	64 ± 15^‡^	88 ± 14	<0.01
GOLD stage (%) (I/II/III-IV)	0/47/54	18/61/21	62/38/0	<0.01^a^
FVC (L)	3.42 ± 0.97	3.53 ± 0.94	4.02 ± 0.78	0.09
FVC (% pred)	93 ± 16^*^	100 ± 14^‡^	113 ± 13	<0.01
FEV_1_/FVC (%)	40 ± 11^*^^†^	49 ± 11^‡^	60 ± 7	<0.01
VC (L)	3.33 ± 0.91^*^	3.33 ± 0.78^‡^	4.05 ± 0.81	0.01
VC (% pred)	90 ± 17^*^	97 ± 14^‡^	113 ± 13	<0.01
TLC (L)	6.85 ± 1.71	6.32 ± 1.39	6.54 ± 1.00	0.35
TLC (% pred)	108 ± 17	105 ± 13	107 ± 14	0.64
IC/TLC (%)	36 ± 10^*^	40 ± 11	45 ± 9	<0.01
RV (L)	3.52 ± 1.11^*^	2.98 ± 0.92	2.48 ± 0.50	<0.01
RV (% pred)	154 ± 41^*^	133 ± 32	112 ± 26	<0.01
RV/TLC (%)	51 ± 8^*^	47 ± 7^‡^	38 ± 7	<0.01
FRC (L)	4.65 ± 1.33^*^	4.03 ± 1.21	3.66 ± 0.75	0.01
FRC (% pred)	147 ± 32^*^	132 ± 29	117 ± 24	<0.01
D_LCO_ (ml/mmHg/min)	14.5 ± 4.9	16.1 ± 5.2	18.2 ± 6.4	0.11
D_LCO_ (% pred)	63 ± 18^*^	72 ± 19	79 ± 21	<0.01
D_LCO_/V_A_ (ml/mmHg/min)	3.25 ± 0.91	3.60 ± 0.84	3.43 ± 0.81	0.23
D_LCO_/V_A_ (% pred)	79 ± 22	86 ± 19	82 ± 19	0.27
*Medications [n (%)]*				
SABA	28 (61)	18 (55)	7 (44)	
Anticholinergic	28 (61)	15 (45)	4 (25)	
LABA/LAMA	12 (26)	1 (3)	0 (0)	
ICS/LABA	21 (46)	16 (48)	4 (25)	
Inhaled corticosteroid	6 (13)	3 (9)	1 (6)	
Statin	10 (22)	3 (9)	6 (4)	
ARBs	7 (15)	4 (12)	4 (25)	
ACE inhibitor	5 (11)	6 (18)	0 (0)	
Diuretic	6 (13)	6 (18)	0 (0)	

MRC dyspnea score, measured with the medical research council breathlessness scale; FEV_1,_ forced expiratory volume in 1 s; FVC, forced vital capacity; VC, vital capacity; TLC, total lung capacity; RV, residual volume; FRC, functional residual capacity; D_LCO_, diffusion capacity of the lungs for carbon monoxide; D_LCO_/V_A_, diffusion capacity of the lungs for carbon monoxide corrected for alveolar ventilation. SABA, short-acting β_2_-adrenergic receptor agonist; LABA/LAMA, long-acting β_2_-adrenergic receptor agonist and long-acting muscarinic antagonist; ICS/LABA, inhaled corticosteroid and long-acting β_2_-adrenergic receptor agonist; ARBs, angiotensin II receptor blocker; and ACE inhibitor, angiotensin-converting enzyme inhibitor.

a Value of *p* determined from Chi-Square test.

* Between phenotype comparisons: *p* = 0.05, ventilatory vs. cardiovascular.

† Between phenotype comparisons: *p* = 0.05, ventilatory vs. combined.

‡ Between phenotype comparisons: *p* = 0.05, combined vs. cardiovascular.

### Peak Exercise Responses

**Figure 3:**
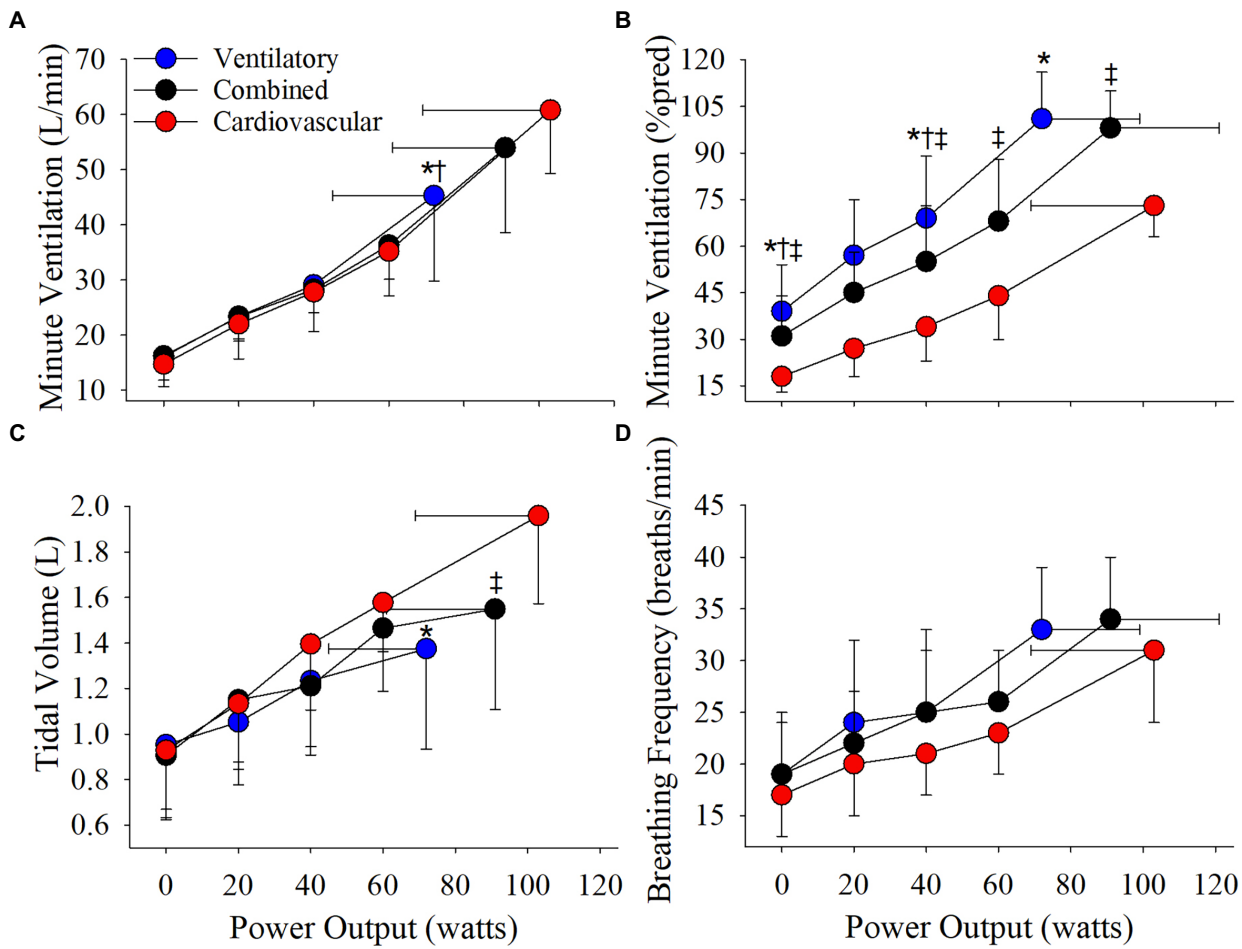
Phenotype responses in **(A)** absolute ventilation, **(B)** relative ventilation (expressed as percentage of estimated MVC), **(C)** tidal volume, and **(D)** breathing frequency during an incremental CPET. Between phenotype comparisons: ^*^p = 0.05, ventilatory vs. cardiovascular. ^†^*p* = 0.05, ventilatory vs. combined. ^‡^*p* = 0.05, combined vs. cardiovascular.

**Figure 4:**
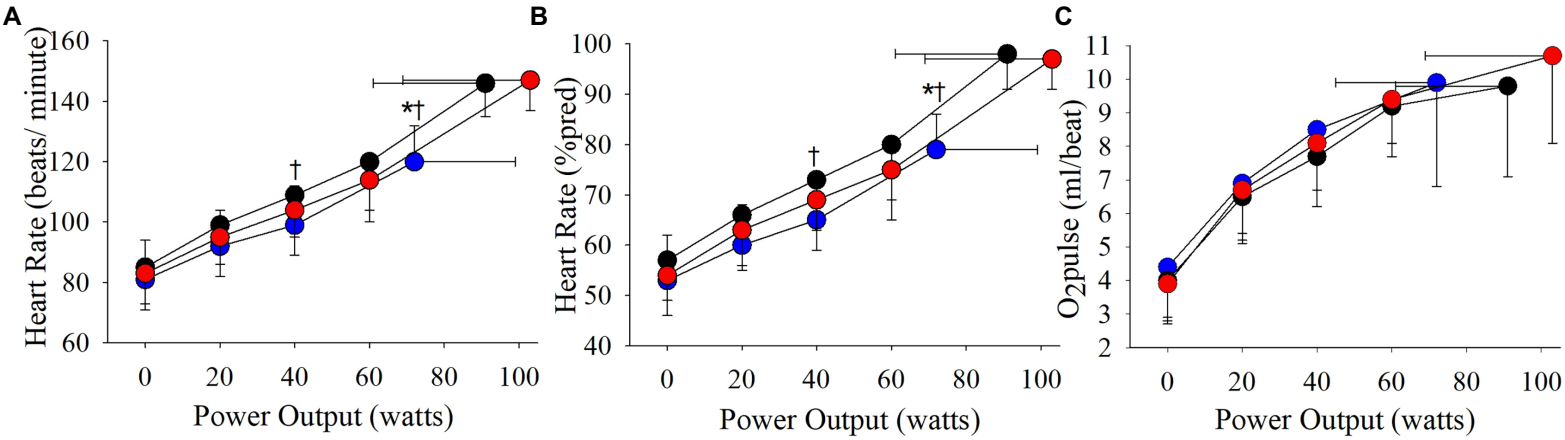
Phenotype responses in **(A)** absolute heart rate, **(B)** relative heart rate (expressed as a percentage of estimated maximal heart rate), and **(C)** O_2_pulse during an incremental CPET. Between phenotype comparisons: ^*^p = 0.05, ventilatory vs. cardiovascular.

**Figure 5:**
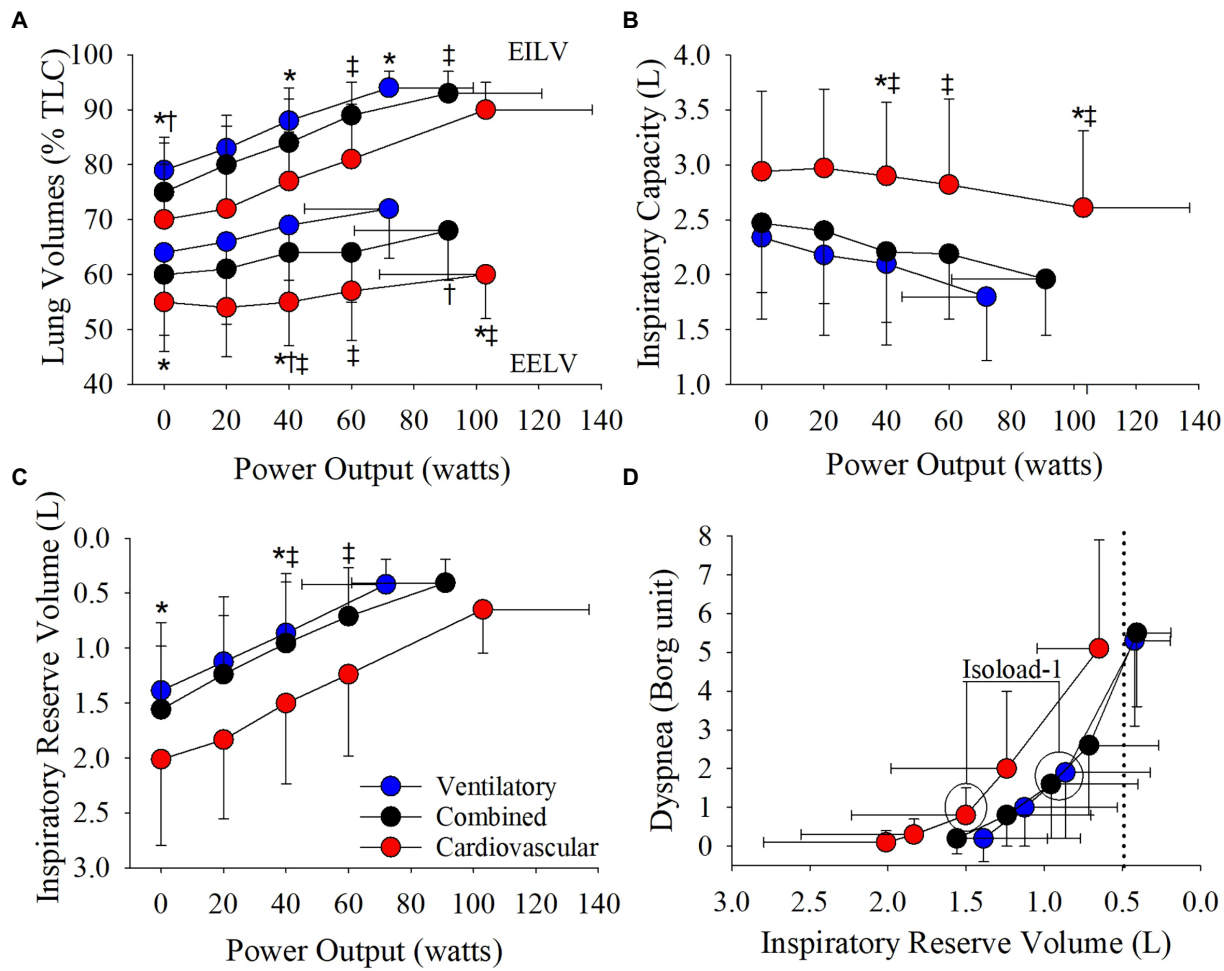
Phenotype responses in **(A)** relative end-expiratory lung volume (EELV) and end-inspiratory lung volume (EILV), **(B)** inspiratory capacity, **(C)** inspiratory reserve volume, and **(D)** the relationship between dyspnea and inspiratory reserve volume during an incremental CPET. Between phenotype comparisons: ^*^p = 0.05, ventilatory vs. cardiovascular. ^†^*p* = 0.05, ventilatory vs. combined. ^‡^*p* = 0.05, combined vs. cardiovascular.

The cardiovascular phenotype reached a higher HR_peak_ and V_Epeak_ compared to the ventilatory phenotype but not the combined phenotype (Table 2; Figures 3, 4). Patients with a cardiovascular phenotype had a larger V_T_ (Figure 3), and lower end-expiratory lung volume (EELV) and EILV compared to both other phenotypes (Figure 5). IC was larger in the cardiovascular phenotype (Figure 5); however, ΔIC was not different between phenotypes (−0.33 ± 0.43 L, −0.51 ± 0.26 L, and −0.54 ± 0.33 L in cardiovascular, combined, and ventilatory, respectively, *p* = 0.09). Inspiratory reserve volume (IRV), V_T_/IC, O_2_pulse, and exertional symptoms were not different between phenotypes (Table 2; Figures 4, 5). Workload, VO_2_, VCO_2_, SpO_2_, and respiratory exchange ratio (RER) were similar between the cardiovascular and combined phenotypes but were lower in the ventilatory phenotype (Table 2).

**Table 2:** Incremental cardiopulmonary exercise testing (CPET) responses between phenotypes.

Variable	Ventilatory (*n* = 46)	Combined (*n* = 33)	Cardiovascular (*n* = 16)	ANOVA *p*-value
V_Epeak_ (L/min)	45.3 ± 15.5^*^^†^	54.0 ± 15.4	60.8 ± 11.5	<0.01
V_Epeak_ (%MVC)	101 ± 15^*^	98 ± 12^‡^	73 ± 10	<0.01
Ventilatory reserve (L/min)	−0.2 ± 5.8^*^	+1.3 ± 5.9^‡^	+24.4 ± 15.8	<0.01
HR_peak_ (beats/min)	120 ± 12^*^^†^	146 ± 11	147 ± 10	<0.01
HR_peak_ (% pred)	79 ± 7^*^^†^	98 ± 7	97 ± 6	<0.01
Cardiac reserve (beats/min)	32 ± 11^*^^†^	3 ± 10	5 ± 9	<0.01
O_2_pulse (ml/beat)	9.9 ± 3.1	9.8 ± 2.7	10.7 ± 2.6	0.57
Maximum workload (watts)	72 ± 27^*^^†^	91 ± 30	103 ± 34	<0.01
VO_2peak_ (ml/kg/min)	15.2 ± 3.3^*^^†^	18.3 ± 4.3	20.6 ± 4.0	<0.01
VO_2peak_ (% pred)^b^	63 ± 19^*^^†^	86 ± 26	87 ± 17	<0.01
VO_2peak_ (L/min)	1.19 ± 0.40^*^^†^	1.43 ± 0.40	1.57 ± 0.40	<0.01
VCO_2_ (L/min)	1.22 ± 0.46^*^^†^	1.53 ± 0.48	1.77 ± 0.45	<0.01
RER	1.02 ± 0.10^*^^†^	1.07 ± 0.09	1.13 ± 0.10	<0.01
V_E_/VCO_2_ peak	38 ± 7	36 ± 5	35 ± 5	0.08
V_E_/VCO_2_ nadir	38 ± 7^*^^†^	34 ± 5	33 ± 5	<0.01
V_E_-VCO_2_ slope	30 ± 6	27 ± 5	28 ± 4	0.13
V_E_-VCO_2_ intercept	8 ± 4	9 ± 3	7 ± 2	0.17
P_ET_O_2_ (mmHg)	102.9 ± 6.8^*^	106.2 ± 6.5	109.9 ± 6.2	<0.01
P_ET_CO_2_ (mmHg)	35.3 ± 4.3	35.3 ± 4.4	34.4 ± 5.2	0.79
V_D_/V_T_	0.28 ± 0.08^*^^†^	0.22 ± 0.06	0.18 ± 0.04	<0.01
EILV (L)	6.25 ± 1.76	5.84 ± 1.40	5.89 ± 0.88	0.40
EILV (%TLC)	94 ± 3^*^	93 ± 4^‡^	90 ± 5	<0.01
EELV (L)	4.88 ± 1.58	4.29 ± 1.36	3.93 ± 0.77	0.04
EELV (%TLC)	72 ± 9^*^^†^	68 ± 9^‡^	60 ± 8	<0.01
IRV (L)	0.42 ± 0.23	0.41 ± 0.22	0.65 ± 0.40	0.04
V_T_/IC (%)	77 ± 9	79 ± 9	77 ± 10	0.49
SpO_2_ (%)	92 ± 4^*^^†^	95 ± 4	96 ± 2	<0.01
ΔSpO_2_ (%)	−3 ± 3^*^	−2 ± 3	−1 ± 2	0.01
Dyspnea (Borg 0–10 scale)	5.3 ± 2.2	5.5 ± 1.9	5.1 ± 2.8	0.76
Leg fatigue (Borg 0–10 scale)	5.4 ± 2.5	5.9 ± 2.5	5.9 ± 2.7	0.69
Dyspnea/LF/Both (%)	39/46/15	42/42/15	31/44/25	0.89^a^

V_Epeak_, peak minute ventilation; MVC, estimated maximum ventilatory capacity; HR_peak_, peak heart rate; VO_2peak_, peak oxygen consumption; VCO_2_, volume of exhaled carbon dioxide; RER, respiratory exchange ratio; V_E_/VCO_2_, ratio of minute ventilation to volume of exhaled carbon dioxide; P_ET_O_2_, partial pressure of end-tidal oxygen; P_ET_CO_2_, partial pressure of end-tidal carbon dioxide; V_D_/V_T_, estimated ratio of dead space ventilation to tidal volume obtained in ventilatory *n* = 33, combined *n* = 26, and cardiovascular *n* = 12; EILV, end-inspiratory lung volume; EELV, end-expiratory lung volume; V_T_/IC ratio of tidal volume to inspiratory capacity; SpO_2_, peripheral oxyhemoglobin saturation; ΔSpO_2_, change in peripheral oxyhemoglobin saturation from rest to peak exercise; LF, leg fatigue; and Both, both dyspnea and leg fatigue.

a Value of *p* determined from Chi-Square test.

b Calculated using the FRIEND database (de Souza e Silva et al., 2018).

* Between phenotype comparisons: *p* = 0.05, ventilatory vs. cardiovascular.

† Between phenotype comparisons: *p* = 0.05, ventilatory vs. combined.

‡ Between phenotype comparisons: *p* = 0.05, combined vs. cardiovascular.

### Submaximal Exercise Responses

Absolute V_E_, V_T_, and breathing frequency were not different between phenotypes at isoloads (Figure 3). However, EELV was lower and IRV was larger in the cardiovascular phenotype compared to both other phenotypes at isoload-1 and the combined phenotype at isoload-2 (Figure 5). EILV was lower in the cardiovascular phenotype vs. ventilatory phenotype at isoload-1 and vs. the combined phenotype at isoload-2 (Figure 5). The cardiovascular phenotype had a larger IC at both isoloads (Figure 5) and a smaller ΔIC at isoload-1 (*p* = 0.02) and isoload-2 (*p* = 0.056) compared to both other phenotypes. V_T_/IC was lower in the cardiovascular phenotype at isoload-1 and isoload-2 compared to the ventilatory (*p* = 0.01) and combined (*p* = 0.02) phenotypes, respectively. Heart rate was lower at isoload-1 in the ventilatory vs. combined phenotype; however, O_2_pulse was not different between phenotypes (Figure 4). V_E_/VCO_2_ nadir was higher in the ventilatory phenotype than both other phenotypes (Table 2). VO_2_ and exertional symptoms were not statistically different between phenotypes.

## Discussion

This study is the first to provide empirical evidence that three distinct exercise limitation phenotypes can be identified in COPD that are associated with different physiological incremental CPET responses, not solely dependent upon airflow limitation severity. In partial support of our hypothesis, patients with a cardiovascular phenotype had the least amount of static hyperinflation and larger IC throughout exercise compared to both other phenotypes. Patients with a cardiovascular phenotype also had a higher VO_2peak_ and W_max_ compared to the ventilatory phenotype but were similar to the combined phenotype.

### Lung Volume Responses to Exercise

Compared to the ventilatory phenotype, the cardiovascular phenotype had less static hyperinflation which allowed a greater reserve for V_T_ expansion resulting in a greater peak V_T_ and V_E_. Additionally, EELV remained lower in the cardiovascular phenotype due to slower dynamic hyperinflation as IC was reduced by ~330 ml over ~100 W compared to ~540 ml over ~70 W in the ventilatory phenotype. In COPD, it has been suggested that a critical inspiratory constraint to V_T_ expansion occurs when IRV reaches 500–600 ml, EILV ≥ 90%TLC and V_T_/IC ~70% (O’Donnell et al., 2006, 2012; Ofir et al., 2008; Laveneziana et al., 2011; Guenette et al., 2014). In the cardiovascular phenotype, IRV was reduced to ~650 ml, EILV reached ~90%TLC and V_T_/IC was ~77% at peak exercise. However, when compared to recently published age-and-sex-matched normative CPET reference equations (Lewthwaite et al., 2020), IRV was >LLN and EILV (%TLC) and V_T_/IC were below the upper limit of normal (ULN) in 14/16 patients with a cardiovascular phenotype. Additionally, peak IC and V_T_ were >LLN in 100% of the cardiovascular phenotype demonstrating normal V_T_ expansion. The cardiovascular phenotype also appeared to exhibit a relatively normal hyperventilatory response after the respiratory compensation point as V_E_/VCO_2_ significantly increased from nadir to peak (33 ± 5 vs. 35 ± 5, *p* < 0.001) and P_ET_CO_2_ significantly decreased (36.6 ± 5.1 mmHg vs. 34.4 ± 5.2 mmHg, *p* < 0.001), while peak RER was >1.10 (Inbar et al., 1994; Neder et al., 2001). These findings taken together with the ability to reach ≥90%pred HR_max_, while maintaining a significant ventilatory reserve at VO_2peak_, demonstrate that the cardiovascular phenotype essentially exhibited a normal ventilatory and cardiovascular exercise response similar to healthy aging. As such, while minor alterations in pulmonary mechanics likely contribute to exercise limitation, they do not appear to be the primary limitation in the cardiovascular phenotype.

In the ventilatory phenotype, greater static and dynamic hyperinflation resulted in V_T_ constraint and reduced peak V_T_ and V_E_ as EILV rose to ~94%TLC and IRV reached ~420 ml at a significantly lower W_max_ compared to both other phenotypes. Breathing at higher lung volumes increases the elastic work of breathing (Eves et al., 2006) and creates an imbalance between the inspiratory neural drive to breathe and ability to efficiently increase V_E_, resulting in intolerable dyspnea and exercise cessation (O’Donnell et al., 2006, 2012; Ofir et al., 2008; Laveneziana et al., 2011; Guenette et al., 2014). At isoload-1, EELV, EILV, and V_T_/IC were lower and IRV was ~175% larger in the cardiovascular vs. ventilatory phenotype. In the cardiovascular phenotype, breathing at lower lung volumes maintained a more efficient breathing pattern that likely contributed to the lower dyspnea at isoload-1 (*p* = 0.03, ANOVA main effect) enabling the cardiovascular phenotype to reach a higher W_max_. In the combined phenotype, EELV was also lower at isoload-1 compared to the ventilatory phenotype. As such, the combined phenotype reached both a ventilatory and cardiovascular limitation at peak exercise, and a higher VO_2peak_, W_max_, and V_Epeak_ compared to the ventilatory phenotype.

While it is acknowledged that, on average, the cardiovascular phenotype had milder airflow obstruction compared to both other phenotypes, the range in FEV_1_ across phenotypes supports that phenotype classification is not solely dependent upon airflow limitation severity. Patients with moderate obstruction were scattered across all three phenotypes (Figure 2) and accounted for ~40% of the cardiovascular phenotype (FEV_1_:74 ± 5%pred) indicating that a subset of patients had similar physiological exercise responses to healthy aging despite having moderate airflow obstruction. Additionally, ~20% of the combined phenotype presented with severe obstruction (FEV_1_:42 ± 8%pred) demonstrating that VO_2peak_ was limited by the attainment of peak cardiac output and convective O_2_ delivery in addition to abnormal ventilatory mechanics.

### Cardiovascular Exercise Responses

In health, VO_2peak_ is predominantly limited by the cardiovascular system due to a finite cardiac output once HR_max_ is reached (Astrand et al., 1964; Higginbotham et al., 1986; Plotnick et al., 1986). In the current study, ~50% of patients reached HR_peak_ ≥ 90%pred, supporting that peak cardiac output and convective O_2_ delivery to the skeletal muscle contribute to exercise limitation in a large percentage of COPD patients. Only one previous study has categorized exercise limitations in COPD to better understand the variable adaptations gained following pulmonary rehabilitation (Plankeel et al., 2005). Utilizing slightly different criteria (i.e., HR_peak_ ≥ 80%pred), a similar percentage of patients (56%) were reported to achieve a cardiovascular limitation with or without a ventilatory limitation (Plankeel et al., 2005). Acknowledging the limitations of O_2_pulse as a surrogate of stroke volume (Whipp et al., 1996), peak O_2_pulse was greater than the LLN (Lewthwaite et al., 2020) in 40/49 patients who reached HR_peak_ ≥ 90%pred, suggesting that the majority of these patients had a normal stroke volume response. Although the O_2_pulse response was not statistically different between phenotypes, peak cardiac output would be expected to be significantly greater in the cardiovascular and combined phenotypes due to reaching a higher HR_peak_, which may partly explain the higher VO_2peak_ achieved compared to the ventilatory phenotype.

### Metabolic Exercise Responses

Fifty-six percentage of the cardiovascular and 48% of the combined phenotype had a normal VO_2peak_ (i.e., VO_2peak_ > 84% of age-and sex-predicted; American Thoracic Society and American College of Chest Physicians, 2003) demonstrating preserved cardiopulmonary fitness in certain individuals. Interestingly, ~25% of the cardiovascular and combined phenotypes reached a VO_2peak_ ≥ 100%pred. Given that the cardiovascular phenotype had a relatively normal ventilatory and peak O_2_pulse response and that the VO_2_-workrate relationship was normal (i.e., >8.5 ml/min/watt; Hansen et al., 1988) in 15/16 patients, it is likely that the low VO_2peak_ reported in the remaining 44% of patients with a cardiovascular phenotype was due to deconditioning. In contrast, 87% of the ventilatory phenotype achieved a VO_2peak_ < 84%pred. V_E_/VCO_2_ nadir was highest in the ventilatory phenotype as exercise cessation occurred at a lower W_max_ (often before the respiratory compensation point) due to V_T_ constraint and greater dead-space. Despite differences in dynamic hyperinflation at isoloads and V_T_ constraint at peak exercise, the V_E_-VCO_2_ slope and intercept did not differ between phenotypes demonstrating that the ventilatory response to VCO_2_ and the CO_2_ set-point were similar and independent of exercise limitation phenotype. In COPD, it has been suggested that an EILV ≥ 90%TLC and a V_E_/VCO_2_ nadir >34 more strongly predicts reductions in VO_2peak_ compared to ventilatory reserve (Neder et al., 2019). In the current study cohort, 82% of all patients reached an EILV ≥ 90%TLC and a V_E_/VCO_2_ nadir >34 varied between phenotypes (67% in ventilatory, 39% in combined, and 50% in cardiovascular). Regardless, VO_2peak_ was significantly higher in the cardiovascular and combined phenotypes vs. the ventilatory phenotype. Therefore, classifying patients phenotypically may be a more appropriate method to predict reductions in an integrative measure like VO_2peak_. Furthermore, the identification of a ventilatory phenotype may be of prognostic importance as VO_2peak_ was below normative values in the majority of these patients (Cote et al., 2008).

### Skeletal Muscle Contributions

It must be acknowledged that all systems in the O_2_ cascade integratively contribute to the body’s inability to meet metabolic demand even in patients with advanced lung disease (Maltais et al., 2000; Puente-Maestu et al., 2009; Broxterman et al., 2020). In COPD, alterations in skeletal muscle structure and function contribute to exercise limitation (Maltais et al., 1996, 2000; Saey et al., 2005; Puente-Maestu et al., 2009). In many patients, skeletal muscle deconditioning and/or dysfunction leads to a greater reliance on anaerobic glycolysis resulting in increased H^+^ and CO_2_ production above the anaerobic threshold (Maltais et al., 1996; Saey et al., 2005). Increased drive to breathe from chemoreceptor stimulation in addition to type III/IV afferents (Gagnon et al., 2012; Bruce et al., 2016) could accelerate dynamic hyperinflation and V_T_ constraint leading to a ventilatory limitation at a lower workload, independent of airflow limitation severity. However, with maintained or improved skeletal muscle quality ventilatory drive is likely reduced allowing heart rate to rise closer to maximal values. Therefore, the ability for certain patients to achieve age-and-sex-predicted VO_2peak_ may be associated with preserved or enhanced skeletal muscle quality.

### Clinical Relevance

Although the submaximal exercise responses varied between the three exercise limitation phenotypes, exercise responses ranged even within phenotype. This is not surprising as many groups have demonstrated that all steps within the O_2_ cascade contribute to VO_2peak_ in health and also in individuals with COPD (Maltais et al., 2000; Richardson et al., 2004; Broxterman et al., 2020). As such, in patients who are predominantly ventilatory limited, cardiac output and systemic O_2_ delivery still contribute to exercise limitation albeit to a smaller degree than abnormal lung mechanics and tidal volume constraint. Similarly, patients with a predominantly cardiovascular limitation are primarily limited by the obtainment of cardiac output and systemic O_2_ delivery but also have a smaller respiratory contribution. Therefore, exercise limitations in COPD likely lie on a continuum with the ventilatory and cardiovascular phenotypes positioned at either end of the continuum separated by the combined phenotype. The transitions between phenotypes demarcate where the pulmonary and/or cardiovascular systems significantly limit VO_2peak_.

As incremental CPET responses differ between phenotypes, the use of a generic exercise prescription even if individualized (i.e., 60%W_max_) will result in different durations (and thus volume) of exercise that can be achieved due to the different ventilatory, cardiovascular, and metabolic responses associated with each exercise limitation phenotype. This may explain previous findings in which patient with COPD who demonstrated a cardiovascular limitation achieved the greatest improvement in VO_2peak_ following pulmonary rehabilitation compared to their ventilatory limited counterparts (Plankeel et al., 2005). Additionally, in the pulmonary rehabilitation setting it may be assumed that the majority of patients with moderate airflow obstruction are primarily ventilatory limited. However, our data demonstrates that patients with moderate airflow limitation represent a significant portion of all three phenotypes (Figure 2). By identifying the patient-specific exercise limitation phenotype, practitioners can prescribe a more appropriate exercise prescription for each patient to target the ventilatory and/or cardiovascular limitation to exercise. With this tailored approach, more patients are likely to gain important physiological adaptations and improvements in clinical outcomes thus increasing the efficacy of pulmonary rehabilitation for patients with COPD.

### Study Considerations

The estimates for predicting MVC and HR_max_ have a number of limitations that have been previously documented (Johnson et al., 1999; Tanaka et al., 2001). However, alternative techniques (e.g., maximum voluntary ventilation maneuver or VECAP method; Johnson et al., 1999) are either inaccurate in COPD or complex to perform and interpret clinically. Additionally, more recent approaches for determining critical inspiratory constraint (i.e., IRV ~500–600 ml, EILV ≥ 90%TLC, and V_T_/IC ~70%; Laveneziana et al., 2011; O’Donnell et al., 2012) may not distinguish between different exercise limitation phenotypes as these values were similar across groups. As such, while predicting MVC and HR_max_ may not be completely optimal, these measures are routinely used in clinical practice to objectively identify adequate or abnormal cardiovascular and breathing reserves as per current ATS/ACCP recommendations (American Thoracic Society and American College of Chest Physicians, 2003). Therefore, we believe that using these estimates still provides considerable utility to identify important phenotypes of exercise limitation in COPD. Exercise responses may have differed had a treadmill been used due to the greater associated metabolic cost and ventilatory demand (Palange et al., 2000). While this would not affect the identification of patients with a ventilatory phenotype, a small percentage of the cardiovascular phenotype may change to a combined phenotype. However, a number of patients with COPD would still maintain a considerable ventilatory reserve and therefore still present with a primary cardiovascular limitation to exercise even on a treadmill.

## Conclusion

Three distinct exercise limitation phenotypes were identified in COPD that were associated with different physiological incremental CPET responses, not solely dependent upon FEV_1_. The cardiovascular system significantly contributed to exercise limitation in ~50% of patients. The relative contribution of the pulmonary and/or cardiovascular systems to VO_2peak_ (and thus phenotype) is likely mediated by skeletal muscle function. Classifying patients phenotypically may be prognostically important and aid in optimizing exercise prescription for rehabilitative purposes.

## Data Availability Statement

The raw data supporting the conclusions of this article will be made available by the authors, without undue reservation.

## Ethics Statement

The studies involving human participants were reviewed and approved by the University of British Columbia Clinical Research Ethics Board and University of Calgary Conjoint Health Research Ethics Board. The patients/participants provided their written informed consent to participate in this study.

## Author Contributions

JG and NE were responsible for study conception and design. Data acquisition and analysis were performed by JG, MH, JS, SW, BM, GA, MR, and NE. Interpretation of data as well as the drafting and revising of the manuscript was performed by JG, MH, JS, SW, BM, GA, JG, GM, MR, JR, and NE. All authors contributed to the article and approved the submitted version.

## Funding

NE is supported by a Michael Smith Foundation for Health Research Clinical Scholar Award (#7085) and a Canadian Foundation for Innovation Infrastructure Grant (#31368).

## Conflict of Interest

The authors declare that the research was conducted in the absence of any commercial or financial relationships that could be construed as a potential conflict of interest.

## Publisher’s Note

All claims expressed in this article are solely those of the authors and do not necessarily represent those of their affiliated organizations, or those of the publisher, the editors and the reviewers. Any product that may be evaluated in this article, or claim that may be made by its manufacturer, is not guaranteed or endorsed by the publisher.
